# Classification of Pepper Seeds by Machine Learning Using Color Filter Array Images

**DOI:** 10.3390/jimaging10020041

**Published:** 2024-01-31

**Authors:** Kani Djoulde, Boukar Ousman, Abboubakar Hamadjam, Laurent Bitjoka, Clergé Tchiegang

**Affiliations:** 1 Laboratory of Analysis, Simulations and Tests (LASE), Department of Computer Engineering, University Institute of Technology, The University of Ngaoundéré, Ngaoundéré P.O. Box 455, Cameroon; kaniramand@gmail.com; 2 Laboratory of Energy, Signal, Imaging and Automation (LESIA), Department of Electrical Engineering, Energetics and Automatics, National Higher School of Agro-Industrial Sciences, The University of Ngaoundéré, Ngaoundéré P.O. Box 455, Cameroon; boukarousman@gmail.com (B.O.); bitjokalaurent@gmail.com (L.B.); 3 Laboratory of Bioprocesses (LBP), Department of Food Engineering and Quality Control, University Institute of Technology, The University of Ngaoundéré, Ngaoundéré P.O. Box 455, Cameroon; tclerge@yahoo.fr

**Keywords:** Penja, *Piper nigrum*, CFA images, image processing, classification, machine learning

## Abstract

The purpose of this work is to classify pepper seeds using color filter array (CFA) images. This study focused specifically on Penja pepper, which is found in the Litoral region of Cameroon and is a type of *Piper nigrum*. India and Brazil are the largest producers of this variety of pepper, although the production of Penja pepper is not as significant in terms of quantity compared to other major producers. However, it is still highly sought after and one of the most expensive types of pepper on the market. It can be difficult for humans to distinguish between different types of peppers based solely on the appearance of their seeds. To address this challenge, we collected 5618 samples of white and black Penja pepper and other varieties for classification using image processing and a supervised machine learning method. We extracted 18 attributes from the images and trained them in four different models. The most successful model was the support vector machine (SVM), which achieved an accuracy of 0.87, a precision of 0.874, a recall of 0.873, and an F1-score of 0.874.

## 1. Introduction

Pepper is a spice obtained from the berries of different species of pepper plants that belong to the botanical family *Piperaceae*. It is important to differentiate between genuine and counterfeit pepper, as only the fruits of *Piper nigrum*, *Piper cubeba*, and *Piper longum* are legally recognized as “pepper” [1].

The species Piper nigrum produces green, white, or black pepper, depending on the stage of its harvest and the method of preparation. The species *Piper longum* produces long pepper, which was widely used in the Middle Ages but has become almost forgotten today. *Piper cubeba* produces cubeb pepper, which is round and has a small tail, hence its name, “tailed pepper”.

According to data from [1], the top five pepper-producing countries were Vietnam, Brazil, Indonesia, India, and China. Vietnam was the largest producer, with a production of 482,977 tons, followed by Brazil, with 113,374 tons, and Indonesia, with 105,817 tons. India and China also produce significant amounts of pepper, with 81,958 and 68,000 tons, respectively. 

Black pepper is traditionally used for its anti-inflammatory properties. Several studies have been carried out in this area, showing a targeted effect of piperine. In particular, it is thought to act by reducing the number of messengers responsible for inflammation in cells affected by osteoarthritis (joint disorders leading to joint pain) [2]. When taken as a course of treatment, it is also thought to reduce pain [3,4]. Laboratory studies have shown the protective effect of piperine on damage linked to oxidative stress. It is also thought to have a beneficial effect on antioxidant enzymes, increasing our protection against oxidation and premature cell ageing [5,6].

In any case, it can be very challenging to distinguish between different types of pepper based on their seeds, as they have similar morphologies [7,8]. This creates a problem of mislabeling on the market. This is the case for Penja pepper in Cameroon, which is one of the rare and exceptional varieties of *Piper nigrum* and is highly coveted by top chefs and gourmands. Its superior quality is due to the unique terroir of Penja, which offers exceptional soil and climate conditions, as well as the specialized knowledge and expertise of the local craftsmen. 

Computer vision, specifically image processing, is a non-destructive testing solution that can be used to address classification problems. The methods employed include machine learning and deep learning, among others. Several studies have already been conducted in this area for spices, with a particular focus on the classification of pepper and chili seeds. For example, in [9], fuzzy logic is used to classify chili and bell pepper seeds. In this study, the accuracy achieved was 85%. The same study was repeated in [7] using 23 different machine learning algorithms. The algorithms that achieved 100% accuracy were Fine KNN, Weighted KNN, Boosted Trees, Bagged Trees, and Subspace KNN. 

Another study was conducted by Awang Iskandar and his team on the detection of foreign bodies in a sample of *Piper nigrum* pepper seeds [8]. They were able to detect foreign bodies, such as pebbles and strings, with 100% accuracy. They employed several segmentation techniques, including the Color and Erodes Segmentation Technique, Color Erode and Clarify Segmentation Technique, and Color and Texture Segmentation Technique. The most effective method was found to be the Color and Texture Segmentation Technique. 

Several studies have demonstrated that the use of color filter array CFA images yields improved results for both segmentation and classification. CFA data are obtained from monochromatic cameras, where the color filter array (CFA) makes each photosensor sensitive to only one color component. CFA images must be demosaiced to obtain the final color images, but this process can negatively impact textural information. This is because demosaicing affects color texture descriptors such as chromatic co-occurrence matrices (CCMs) [10]. A more recent work carried out an analysis of automatic image classification methods for Urticaceae pollen. This work compared machine learning and deep learning methods to classify Urticaceae pollen seeds. It is a very interesting work that shows the power of machine learning and deep learning algorithms in the classification of objects from images [11]. 

This work aimed to improve the authenticity of the product on the market and reduce the problem of usurped labeling. By creating a model that can accurately classify Penja pepper seeds from others, the industry can ensure that consumers are getting the product that they are paying for. Additionally, this work will contribute to the protection of the exceptional terroir of Penja and the know-how of the local craftsmen by making it easier to identify real Penja pepper seeds. This can help to support the local economy and promote sustainable agriculture practices.

The main contributions are listed as follows:

- The creation of a large CFA image database;
- The improvement of the experimental set-up used by Bitjoka et al., 2015 [12];
- The segmentation extraction and attribute extraction method, which can be used for the automation of seed identification in general.

The rest of the work is organized as follows: in Section 2, we first present related works on seed classification. Section 3 is devoted to our samples and image acquisition processes. Then, we show the different classification methods used as well as the selected attributes. In Section 4, we present the results and discussions. The paper is concluded in Section 5.

## 2. Related Work

Several works have been carried out on classification in the field of agri-food, and in relation to spices, classification work has mostly been carried out on peppers and chili peppers. Almost no classification work has been carried out on pepper seeds. However, the techniques and methods used for other spices can also be applied to pepper seeds, the usefulness of which is no longer proven, particularly in the health and culinary fields.

One of the works on pepper seed classification using machine vision is based on convolutional neural networks (CNNs) [13]. In this work, the best classification score of 84.94 precision was achieved with the equipment used: a desktop scanner with a resolution of 1200 dpi. The use of the material can be justified by the fact that the Chili pepper are flat in appearance. Due to not having this material at our disposal, we were not able to reproduce the approach adopted in this work. However, this work clearly shows that neural networks are an effective means of classifying spices.

Another work was carried out on corn seeds [14]. This work focused on the classification of five maize species using computer-based recognition. The models used are Multilayer perceptron (MLP), decision tree (DT), linear discrimination (LDA), naive Bayes (NB), support vector machine (SVM), and k-nearest neighbors (KNN), and the one which yielded the greatest performance was the SVM. These classification models have also been used in several classification projects in the agri-food sector. These works [15,16,17] and many others have shown the effectiveness of these models. As peppercorns have almost the same structure as corn seeds, it is also possible that these methods can work in identifying pepper seeds.

In the food industry, the attributes extracted from a product image directly convey information about the state of the product in the image [18]. To make a classification, it is important to carefully choose the attributes that will serve as elements of comparison in the chosen model. Several works in the literature show that attributes are often selected in terms of shape, color, and texture attributes [19,20,21,22,23]. Among the different texture analysis approaches used in the food industry, the majority of applications use either histograms of sums and differences or chromatic co-occurrence matrices.

Regarding classification performance evaluation methods, several measures have been used in the literature. The most used measures are mentioned in the review [24,25], and there are the following:

- Precision measures the proportion of positive instances correctly identified among all positive instances. It is calculated by dividing the number of true positives by the sum of true positives and false positives.
- Recall measures the proportion of correctly identified positive instances among all truly positive instances. It is calculated by dividing the number of true positives by the sum of true positives and false negatives.
- F-measure, also known as F1 measure, represents a harmonic average of precision and recall. It provides a balanced measure between the two. It is calculated using the formula F1 = 2 × (precision × recall)/(precision + recall).
- Accuracy measures the proportion of correctly classified instances among all instances. It is calculated by dividing the total number of correct predictions by the total number of instances.
- Confusion matrix summarizes the performance of a model in terms of true positives, true negatives, false positives and false negatives. It can be used to calculate other metrics such as precision, recall, and accuracy.

In [26], Sabanci et al. (2022) worked on the classification of Chili Pepper seeds using convolutional neural networks (CNNs). Although their objectives are the same as ours, we worked on pepper seeds, which have a round appearance compared with Chili Pepper seeds, which are rather flat. The device used in the work of [13] for Chili Pepper seeds. This device is well suited for Chili Pepper seeds and not for pepper seeds. The accuracy of the results is well related to the equipment and the size of the database used.

## 3. Materials and Methods

### 3.1. Sample and Image Preparation

**Figure 1:**
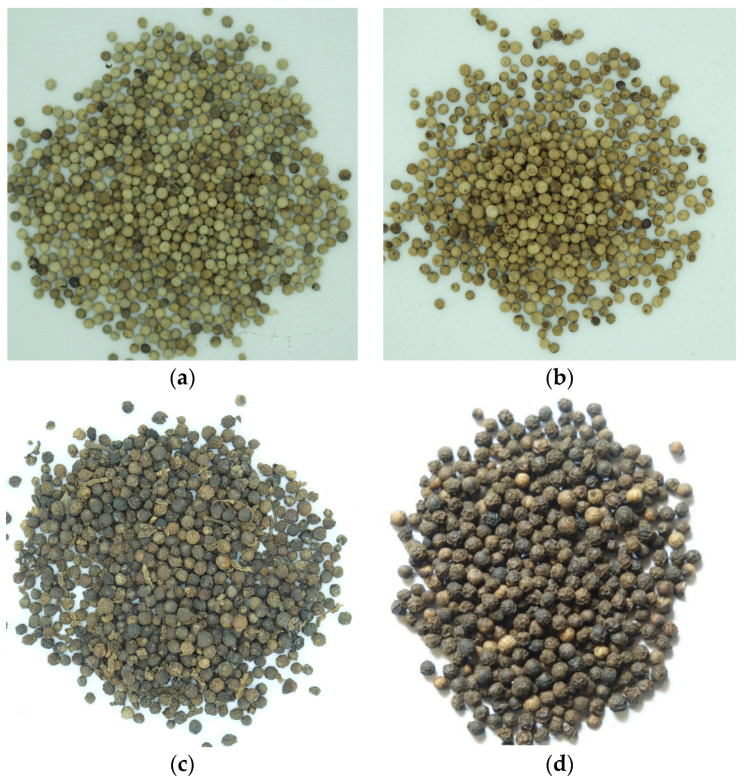
Some samples representing the 4 groups of pepper used: (**a**) white Penja, (**b**) white mixture, (**c**) black Penja, and (**d**) black mixture.

We used both white and black pepper seeds. The samples were divided into four groups: white seeds from Penja, black seeds from Penja, white seeds from other origins, and black seeds from other origins, as presented in Figure 1. The Penja pepper seeds were directly obtained from eight different sources in Penja, resulting in eight distinct samples of Penja pepper, five of which were white and three were black. The other origins comprised a mixture of peppers imported into Cameroon, such as those from Dubai, India, and Brazil.

The different sources of the pepper root samples used are described in Table 1.

### 3.2. Images Acquisition Device

**Figure 2:**
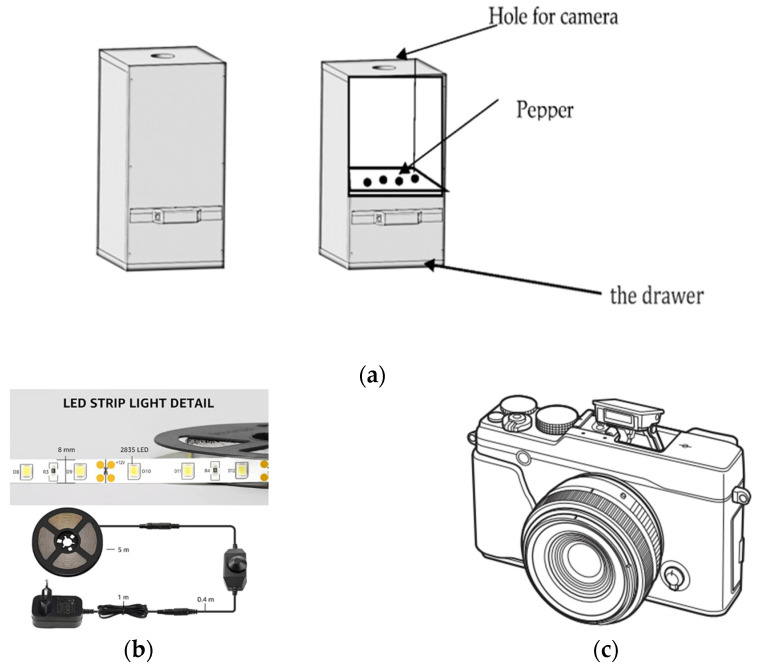
Image capture equipment: (**a**) the box, (**b**) LED strip Light, and (**c**) the Fujifilm X-E1 digital camera [27,28].

We used a device similar to the one used in [12]. This dispositive was established in the Mechanic laboratory of the University Institute of Technology of Brest. Figure 2 shows (a) the image-taking box, (b) the light source, and (c) the camera used.

This box is made of wood and is sealed off from external light. The only source of light is the 150 W LED ribbon on the top inside and the intensity of the light can be adjusted using a potentiometer. 

The Fujifilm X-E1 (Amazon France, Brest, France) digital camera was selected for taking images because its high resolution and good image quality. The images were taken with a resolution of 4896 × 3264 pixels. The aperture was set to f/8, the ISO was set to 400, and the shutter speed was set to 1/60 s to ensure that the images captured had good depth of field, low noise, and good sharpness. The images were taken in RAW format in 14 Bit and later converted to the PGM format for further processing. The device was tested and validated by a team. This device has been used in the following way

➢ The drawer is placed inside the lightproof box
➢ The camera is positioned above the drawer and focused on the seeds
➢ The image is captured with the camera

This process is repeated for 10 pinches of the same sample. The images are taken in RAW (.RAF) + JPG (1920 × 1280 pixels, Size: 24.9 Mb, No flash).

### 3.3. Creation of the Dataset

**Figure 3:**
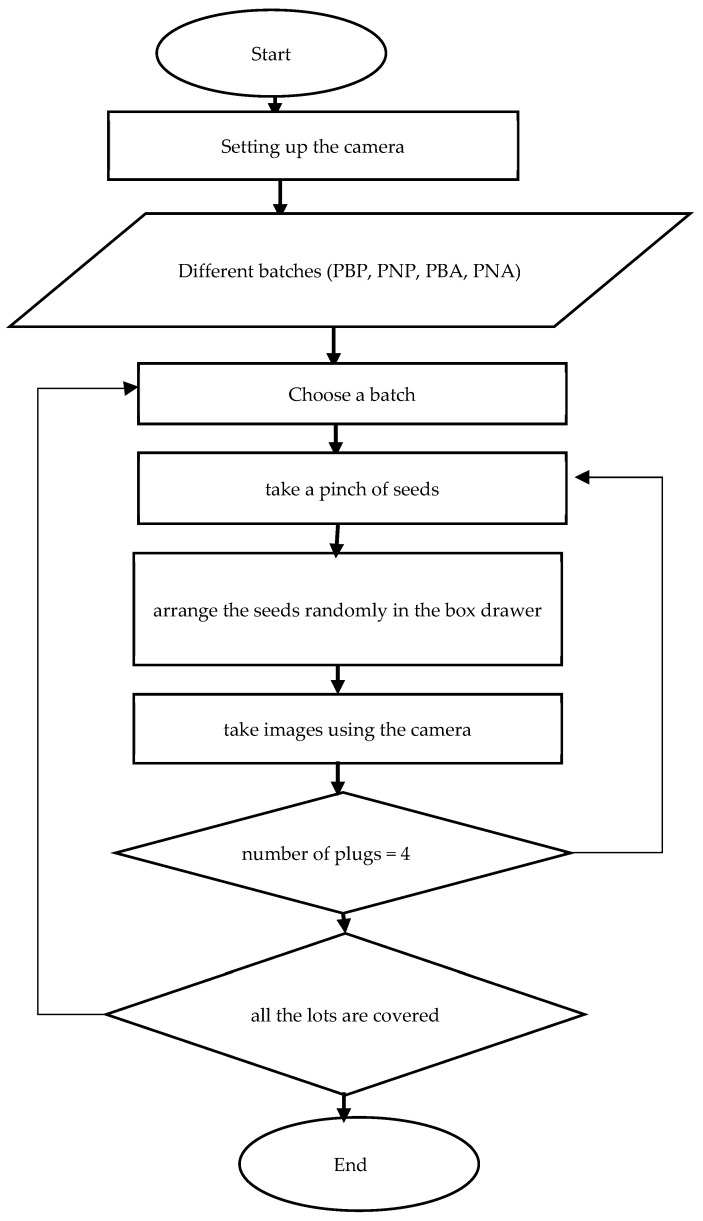
Image acquisition flowchart.

With the Python library *rawpy*, (Python 3.9.13, Anaconda environment, jupyterLab 3.4.4) we generated a 16-bit grayscale image with the PGM (Portable Graymap) extension. The flowchart describing the procedure for creating image data for classification is shown in the diagram below in Figure 3.

**Figure 4:**
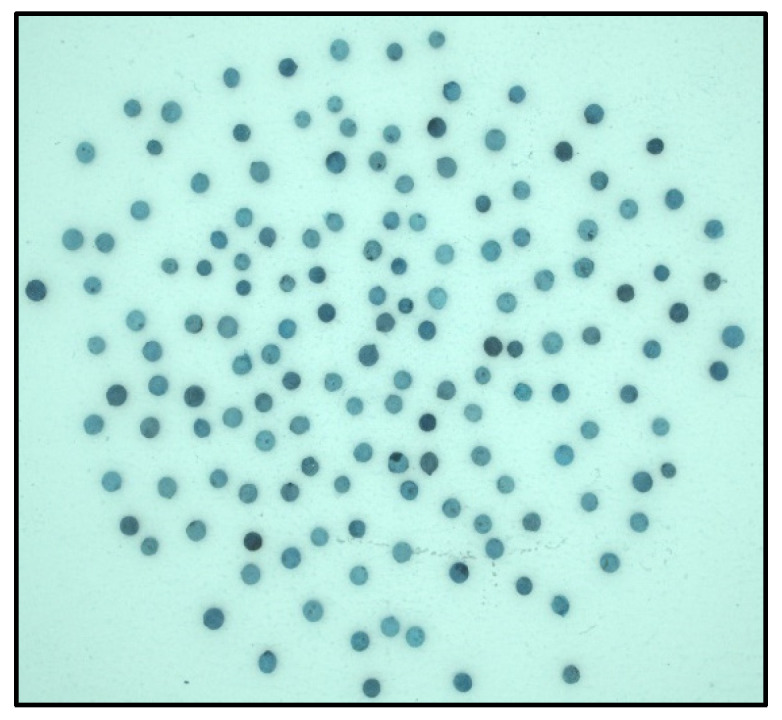
Grayscale image (PGM).

**Figure 5:**
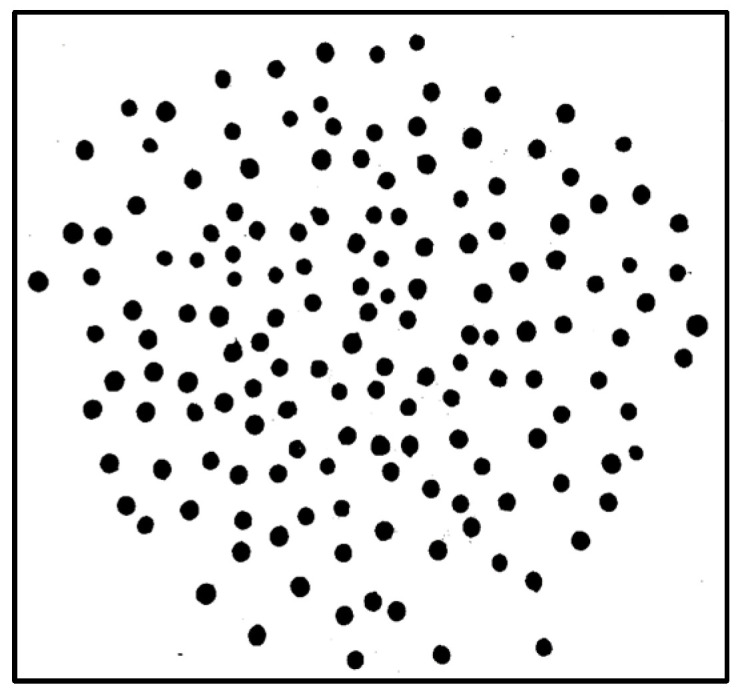
Binary mask.

**Figure 6:**
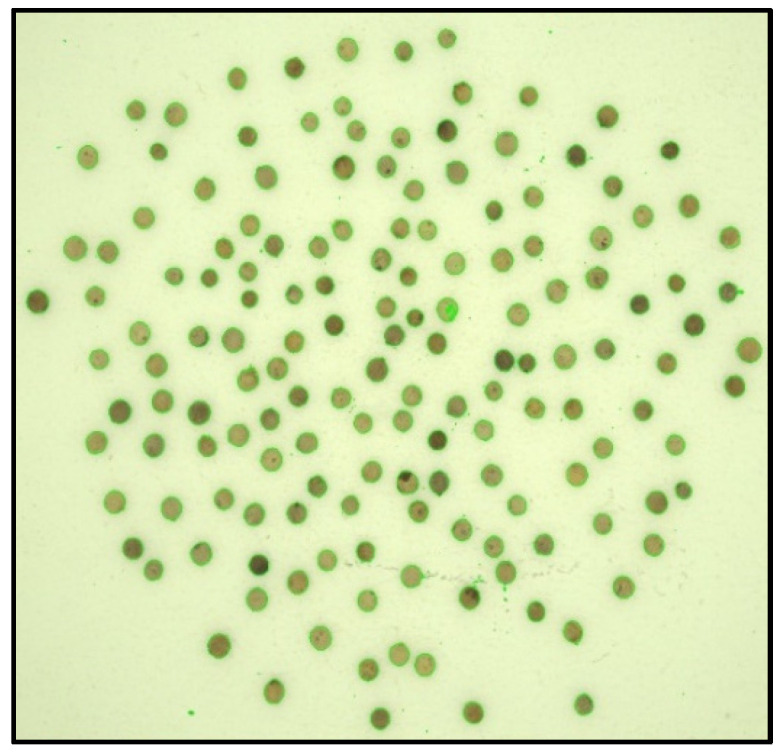
Seed detection using the mask.

**Figure 7:**
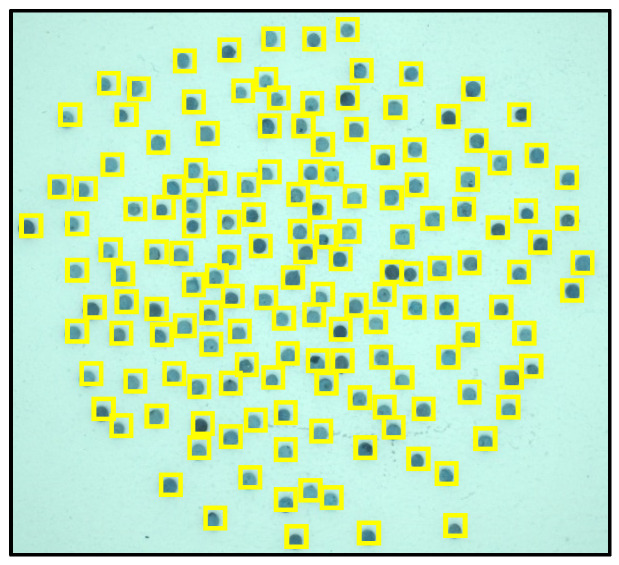
Creation of the Bounding Boxes.

**Figure 8:**
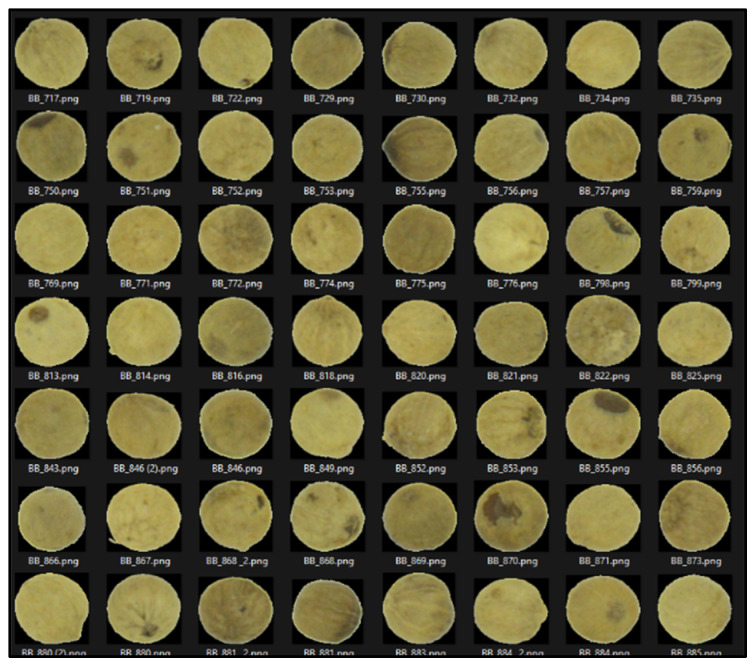
Images of extracted seeds.

These CFA grayscale images (Figure 4) were then segmented using the Otsu method, allowing us to create binary masks (Figure 5) to extract the seeds. Using the masks, we identified each seed (Figure 6) and then created a Bounding Box (smallest quadrilateral that contains the detected object) around each seed (Figure 7) on the CFA image. Finally, we saved the image of each seed in a PNG file (Figure 8). 

The extracted images were stored in 4 different folders, which would constitute our different code classes: PBP (for white pepper from Penja), PBA (for white pepper from other origins), PNP (for black pepper from Penja), and PNA (for black pepper from other origins). We had a total of 5618 seed images: 1335 were PBP, 1416 were PBA, 1437 were PNP, and 1430 were PNA.

Machine learning methods require the manual selection of relevant features prior to extracting them from the images. One challenge lies in the appropriate selection of a set of features for classification [6]. The attributes retained for calculation on each seed image are primarily texture attributes, as texture is an important characteristic used in identifying objects or regions of interest in an image, whether it be a photomicrograph, aerial photograph, or satellite image [29].

The images attributes used can be grouped into 4 main groups like in [29,30]:

- Shape attributes: area, perimeter, compactness, extent, width, and height;
- The characteristics of the Gabor filter: the mean and the standard deviation;
- The characteristics of the LBP (Local Binary Patterns transform: contrast, correlation, energy, homogeneity, and entropy);
- The characteristics of the co-occurrence matrix (GLCM): dissimilarity, correlation, contrast, homogeneity, and ASM.

**Figure 9:**
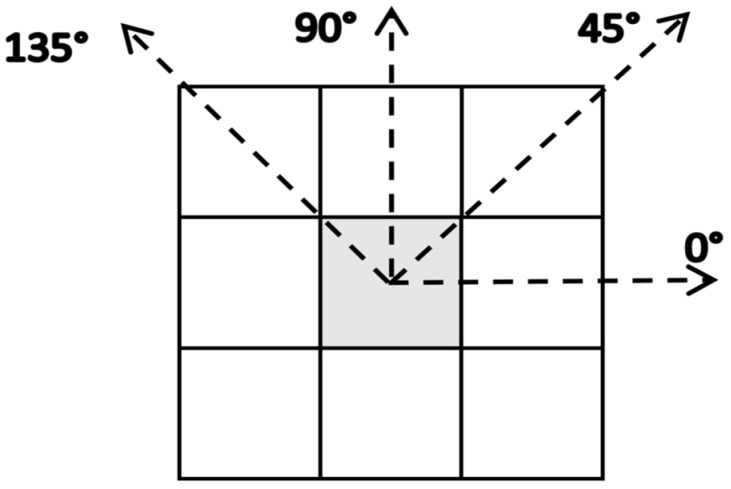
Connected neighbors pixels Grey Level Co-occurrence Matrix [29].

Following [29], a Grey Level Co-occurrence Matrix (GLCM) has been created using neighboring grey tones (Figure 3) in order to derive the textural features. GLCM gives an indication of the spatial relationship of pixels and characterizes the texture of an image by calculating how often pairs of pixels with specific values and in an unambiguous spatial relationship occur in an image. Specifically, GLCM contains the normalized relative frequency, p(i, j), indicating how often two pixels with grey levels i and j separated by a distance d along the angle θ occur within an image block. The separation distance d has been assumed to be d = 1, while the angles are assumed to be θ = 0°, 45°, 90°, and 135°. This is illustrated in Figure 9.

**Figure 10:**
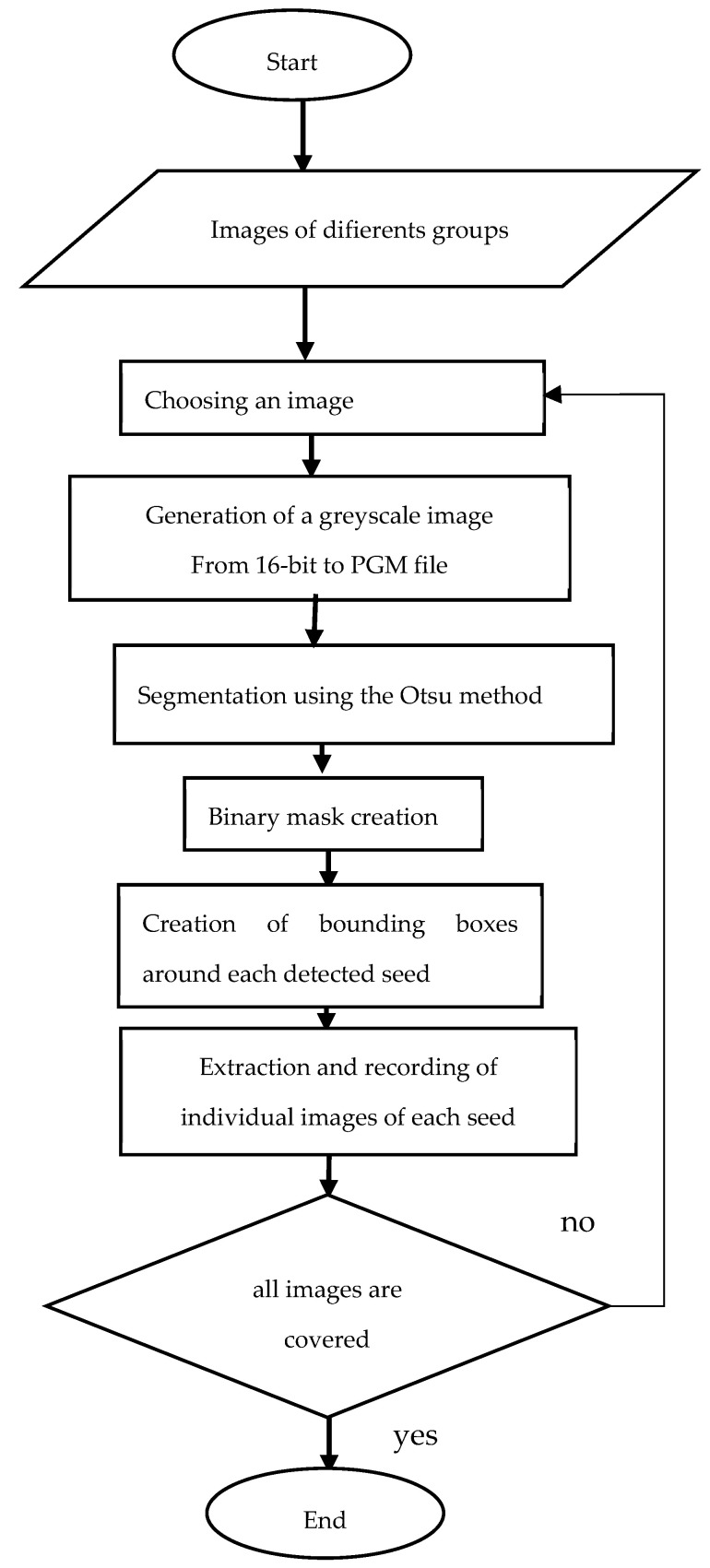
Image attribute extraction flowchart.

The co-occurrence matrix was calculated with a distance of 5 pixels and an angle of 0 degrees. These data are then saved in an Excel file. The feature extraction process for the pepper seed images is described by the following diagram in Figure 10:

### 3.4. Classification

**Figure 11:**
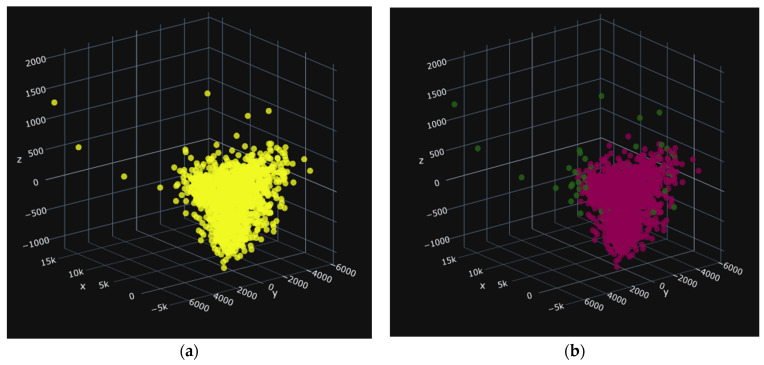
PBP: 1281 data items, i.e., 54 deleted. (**a**) before and (**b**) after.

Before building the different models for classification, we first eliminated outliers using the Isolation Forest algorithm from the sklearn library in Python3.9, with a contamination rate of 0.05 [26]. This process allowed us to remove 282 data points, reducing the number of outliers from 5618 to 5336. The presence of outliers can be attributed to the high variance in shape variables such as area and perimeter. The results of the cleaning process are shown in the following diagrams. Figure 11 shows (a) the distribution of values for the three main attributes on the PBP batches and (b) the cleaning of outliers.

**Figure 12:**
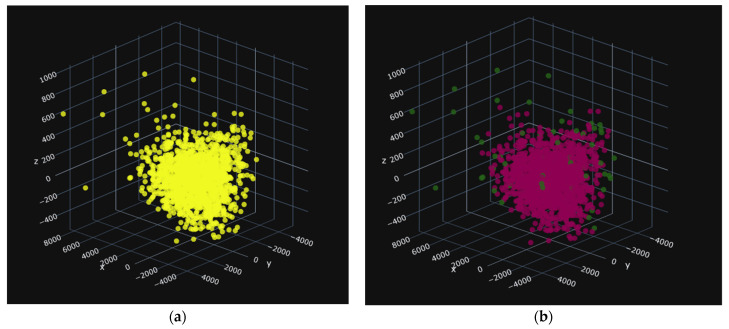
PBA: 1359 data items, i.e., 57 deleted. (**a**) before and (**b**) after.

**Figure 13:**
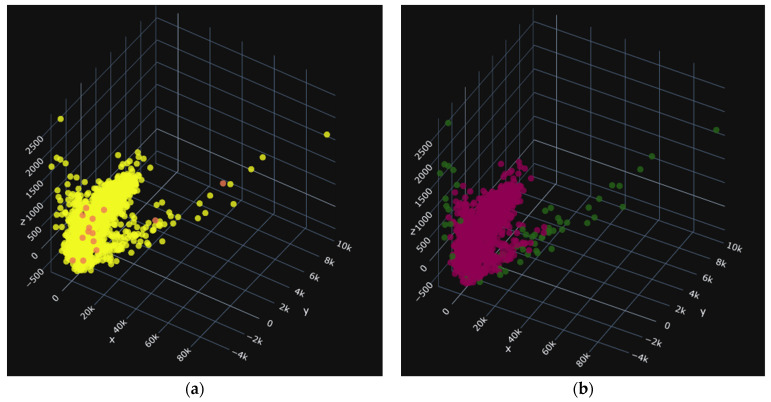
PNP: 1372 données soit 58 supprimées. (**a**) before and (**b**) after.

**Figure 14:**
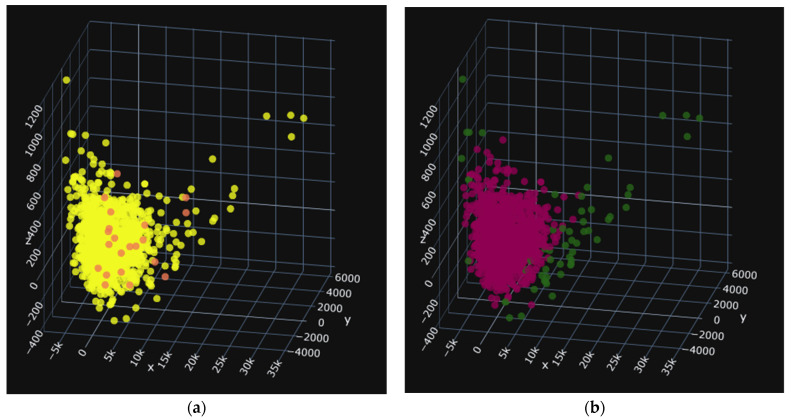
PNA: 1379 données soit 58 supprimées. (**a**) before and (**b**) after.

In these images, the images before cleaning (a), the yellow color, represent all of the seeds in the treated group. In the images after cleaning in Figure 12b, Figure 13b, and Figure 14b the red seeds represent the retained seeds, and those in green are the outliers. The red seeds shown in Figure 13a and Figure 14a represent those that were not recognized.

Figure 12 shows (a) the distribution of values for the three main attributes on the PBA batches and (b) the cleaning of outliers.

Figure 13 shows (a) the distribution of values for the three main attributes on the PBP batches and (b) the cleaning of outliers.

Figure 14 shows (a) the distribution of values for the three main attributes on the PNA batches and (b) the cleaning of outliers.

Next, we normalized the data using RobustScaler algorithm [26]. This scaler removes the median and scales the data based on the quantile range (by default, it uses the interquartile range or IQR). The IQR is the range between the first quartile (25th quantile) and the third quartile (75th quantile). Standardization of a dataset is a common requirement for many machine learning algorithms. Normally, this is carried out by removing the mean and scaling to unit variance, but outliers can negatively impact the sample mean and variance. In such cases, the median and the interquartile range often provide better results. The list of texture attributes used in the literature are present in Table 2, and the feature selection is shown in Table 3.

According to the co-occurrence matrix *P*(*i*, *j*|*d*, *θ*) {*P*_0_(*i*, *j*|*d*)}_NG×NG_ (where NG is in greyscale), we can define many texture features. Reference [29] defined 14 texture features, mainly in the following:

(a) Energy: $E\;\left(P_{0}\left(d\right)\right)=\sum_{i=0}^{N_{G}-1}\sum_{j=0}^{N_{G}-1}{\left[P_{0}\left(i,j|\;d\right)\right]}^{2}$;
(b) Entropy: $H\;\left(P_{0}\left(d\right)\right)=-\sum_{i=0}^{N_{G}-1}\sum_{j=0}^{N_{G}-1}P_{0}\left(i,j|\;d\right)\log P_{0}P_{0}\left(i,j|\;d\right)$;
(c) Correlation: $C\;\left(P_{0}\left(d\right)\right)=\frac{\left[\;\sum_{i=0}^{N_{G}-1}\sum_{j=0}^{N_{G}-1}\left(i-\mu_{x}\right)\left(j-\mu_{y}\right)P_{0}\left(i,j|\;d\right)\right]}{\sigma_{x}\sigma_{y}}$;
(d) Local uniformity: $L\left(P_{0}\left(d\right)\right)=\sum_{i=0}^{N_{G}-1}\sum_{j=0}^{N_{G}-1}\frac{1}{{H\left(i-j\right)}^{2}}P_{0}\left(i,j|\;d\right)$;
(e) Moment of inertia: $I\left(P_{0}\left(d\right)\right)=\sum_{i=0}^{N_{G}-1}\sum_{j=0}^{N_{G}-1}{\left(i-j\right)}^{2}\;P_{0}\left(i,j|\;d\right)$;

$\mu_{x}=\sum_{i=0}^{N_{G}-1}i\sum_{i=0}^{N_{G}}P_{0}(i,j|d)$, $\mu_{y}=\sum_{j=0}^{N_{G}-1}j\sum_{i=0}^{N_{G}}P_{0}(i,j|d)$, $\sigma_{x}^{2}=\sum_{j=0}^{N_{G}-1}{\left(i-\mu_{x}\right)}^{2}\sum_{j=0}^{N_{G}-1}P_{0}\left(i,j|d\right)$.

The co-occurrence matrix is one of the most common methods used in texture analysis. It indicates the interrelationship between greyscale patterns, which are unaffected by the monotonic greyscale transformation. The specific implementation steps for Haralick texture extraction are as follows:

Step 1: Read the image. If the original input is a color image, convert the RGB image to grey to calculate the greyscale co-occurrence matrix in the next step. 

Step 2: The complexity of the greyscale co-occurrence matrix is very high. If the original image has a high greyscale value, we can first compress the greyscale value to reduce the greyscale.

Step 3: Select the distance and angle, then calculate the greyscale co-occurrence matrix. 

Step 4: Select the appropriate textured features, then calculate the texture parameters. 

Step 5: Features can be extracted as required, such as mean and variance, and selected as the final image features.

After selection, we see that the three variable selection algorithms, Variance threshold, Recursive Feature Elimination with cross-validation (RFECV), and ANOVA, take all variables without assigning importance. We have to perform the classifications in three steps: first, with the 5 variables determined via the chi-square test, then with the 10 variables selected via the SGD classifier, and finally, with all the variables.

**Figure 15:**
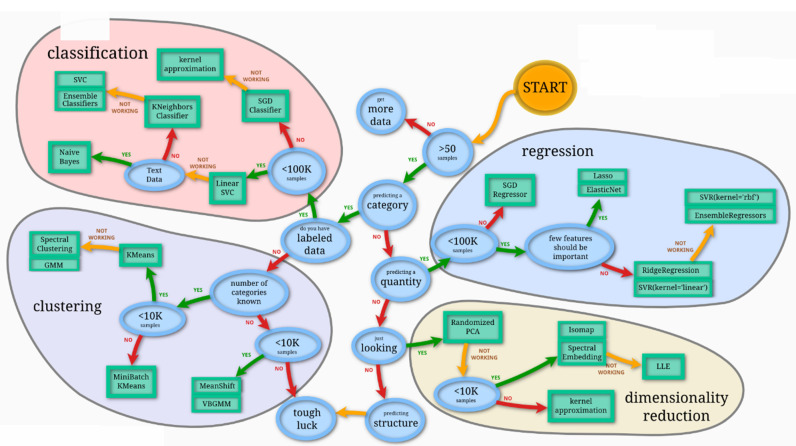
scikit-learn algorithm for choosing the appropriate method [26].

After analyzing the different performances, we found that the best results were obtained by using all the variables. The results of the accuracies, confusion matrices, and learning curves below were obtained, and the results are presented in Section 3. We undertook the classification with the following models: the KNeighbors classifier (KN), SGD classifier, support vector machine (SVM), and random forest (RF). The classification methods were chosen using the scikit-learn algorithm, as shown in Figure 15.

## 4. Results

We have constructed a database of nearly 6000 images of pepper seeds in the RAW format (.RAF), which can be utilized for further research and made accessible to the scientific community. Before proceeding with the training of the models, we divided our data into two parts: 80% (4268) for training and 20% (1068) for testing. For each model, we followed the following steps:

1. Search for optimal parameters with grid search and cross-validation;
2. Train the model with the train set, testing the model with the test set;
3. Construct the confusion matrix;
4. Plot the learning curve.

The models’ performances are present in Table 4.

### 4.1. The Confusion Matrix

**Figure 16:**
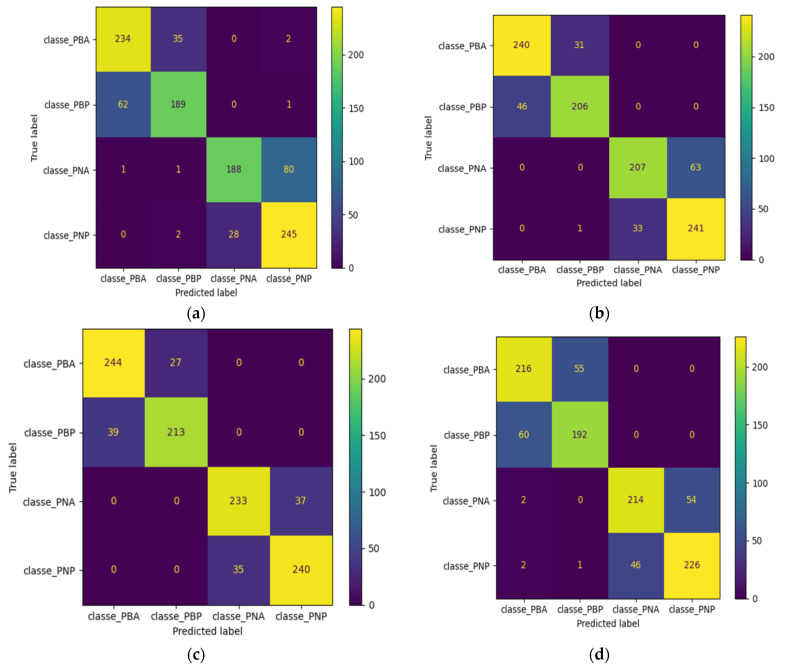
Confusion matrix for the 4 models: (**a**) KNeighbors classifier, (**b**) SGD classifier, (**c**) SVM, and (**d**) random forest.

We notice that the SGD classifier and the SVM are the models that manage to distinguish black pepper seeds from white pepper seeds. The SVM is the model that has the highest accuracy and produces less confusion. In this study, 271 of the white Penja pepper seeds, 86%, were predicted accurately, and 87% of the 270 black Penja pepper seeds were predicted correctly. The Figure 16 present the Confusion matrix for the 4 models used. The Figure 16 present Confusion matrix for the 4 models

### 4.2. Learning Curve

**Figure 17:**
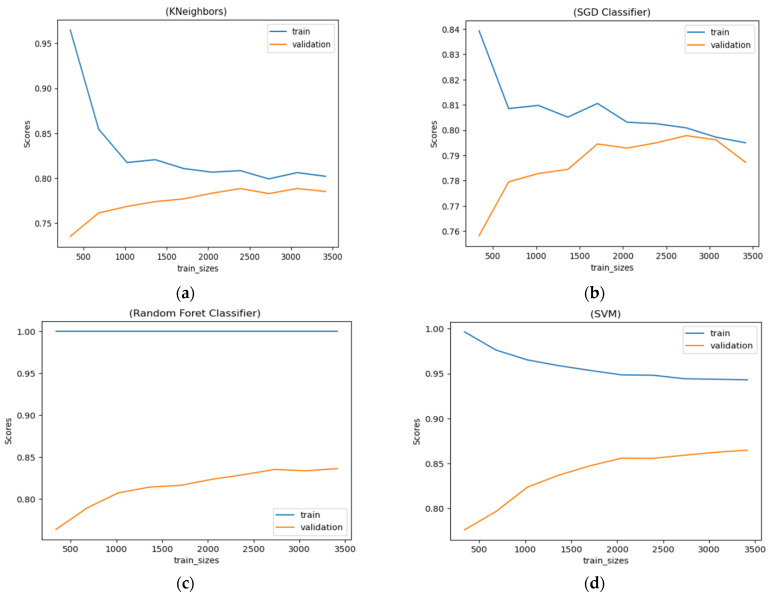
Learning Curve of the 4 methods: (**a**) KNeighbors, (**b**) SGD classifier, (**c**) random forest and (**d**) SMV.

By analyzing the learning curves, we can observe that the random forest model has suffered from overfitting. It fails to generalize well. The SGD model converged around 3100 data points, after which it too experienced overfitting. The KNeighbors and SVM models, however, continue to converge and appear to learn effectively. Hence, the SVM model, which achieved the highest accuracy in classifying pepper seeds, can be considered for use. The Figure 17 present the Learning Curve of the 4 methods.

## 5. Discussion

The classification performance, as described in Table 2, shows that the highest performance achieved is 87. This result can be attributed to the use of shape and size attributes for the classification. Pepper seeds, in general, are similar in appearance and have a similar shape and texture, making them almost indistinguishable to the eye [8]. We worked with 16-bit greyscale images (.pgm) obtained from the sensor’s 14-bit raw RAW data (.RAF). Other similar works use color images, which have already undergone transformations during derrawtisation. Using RAW data gives more information than color images.

A similar study [7] on bell pepper and pimiento seeds produced better results, as the differences between these two species are already visible to the eye. They achieved a score of 89.2 using the SVM and 100 using the KNeighbors and tree classifiers.

## 6. Conclusions

In summary, this study aimed to classify pepper seeds using CFA images. The data used focused on Penja pepper, one of the most coveted in the world, coming from the Litoral region of Cameroon, and achieved an accuracy of 87%. The model was trained on a base of 4268 images, 80% of the data, and tested on 1068, 20% of the data.

Several machine learning methods were employed, and the most successful was the SVM [13]. The precision obtained is higher than that of the [13] same linear SVM method that obtained a precision of 84.94%, which can be justified by the fact that in this work, he used chili seeds, which are almost flat in appearance. 

The method that we used in our work correctly distinguishes between white and black pepper seeds, but there is still some confusion between peppers of the same type. The performance could be improved by using convolutional neural networks, but for that, more image data are required.

## Figures and Tables

**Table 1:** Origin of samples seed.

	Origin 1	Origin 2	Origin 3	Origin 4	Origin 5
Penja white pepper	Penja market seller 1	Penja market seller 2	Penja market seller 3	Penja market seller 4	Penja market seller 5
Penja black pepper	Penja market seller 1	Penja market seller 2	Penja market seller 3	/	/
White pepper mix	Doubaï (Yaoundé super market)	India (Yaoundé super market)	French provinces	/	/
Black pepper blend	Upper Nkam (Yaoundé market)	Black pepper mix (Yaoundé market)	French provinces (Brest supermarket)	/	/

**Table 2:** List of texture attributes used in the literature.

Texturals Features of [29]		
1	Contrast	$\sum_{i=1}^{N_{G}}\sum_{j=1}^{N_{G}}{\left(i-j\right)}^{2}\cdot P(i,j)$
2	Correlation	$\frac{\sum_{i=1}^{N_{G}}\sum_{j=1}^{N_{G}}\left(i-\mu_{x})(j-\mu_{y})\cdot P(i,j\right)}{\sigma_{x}\;\sigma_{y}}$
3	Energy	$\sum_{i=1}^{N_{G}}\sum_{j=1}^{N_{G}}{\left[\;P\left(i,j\right)\;\right]}^{2}$
4	Homogeneity	$\sum_{i=1}^{N_{G}}\sum_{j=1}^{N_{G}}\frac{P\left(i,j\right)\;}{1+{\left(i-j\right)}^{2}}$
5	Sum of squares: variance	$\sum_{i=1}^{N_{G}}\sum_{j=1}^{N_{G}}{\left(i-\mu \right)}^{2}\cdot P(i,j)$
6	Entropy	$-\sum_{i=1}^{N_{G}}\sum_{j=1}^{N_{G}}P(i,j)\cdot \log\;[P\left(i,j\right)]$
7	Sum of averages	$\sum_{k=2}^{2N_{G}}k\cdot P_{x+y}(k)$
8	Entropy sum	$-\sum_{k=2}^{2N_{G}}P_{x+y}(k)\cdot log[P_{x+y}\left(k\right)]$
9	Sum of variance	$\sum_{k=2}^{2N_{G}}{\left(k-\mu_{x+y}\right)}^{2}\cdot P_{x+y}(k)$
10	Difference of variances	$\sum_{k=0}^{N_{G}-1}{\left(k-\mu_{x-y}\right)}^{2}\cdot P_{x-y}(k)$
11	Difference of entropies	$-\sum_{k=0}^{N_{G}-1}P_{x-y}(k)log[P_{x-y}\left(k\right)]$
12	Correlation measure 1 information	HXY−HXY1 / Max(HX,HY)
13	Correlation measure 2 information	${\left[1-exp(-2\cdot HXY2+2\cdot HXY)\;\right]}^{1/2}$
14	Maximum correlation	${\left[Second\;largest\;eigenvalue\;of\;Q\;\;\right]}^{1/2}$
**Texturals features of [31]**		
15	Autocorrelation	$\sum_{i=1}^{N_{G}}\sum_{j=1}^{N_{G}}i\cdot j\cdot P(i,j)$
16	Dissimilarity	$\sum_{i=1}^{N_{G}}\sum_{j=1}^{N_{G}}\left|i-j\right|\cdot P(i,j)$
17	Maximum probability	$Max\;\left(P\left(i,j\right)\right)\;\;\;\;\;\;\;\;\forall \;(i,j)\in (N_{G},\;N_{G})$
18	Cluster nuance	$\sum_{i=1}^{N_{G}}\sum_{j=1}^{N_{G}}{\left(i+j-\mu_{x}-\mu_{y}\;\right)}^{3}\cdot P(i,j)$
19	Cluster prominence	$\sum_{i=1}^{N_{G}}\sum_{j=1}^{N_{G}}{\left(i+j-\mu_{x}-\mu_{y}\;\right)}^{4}\cdot P(i,j)$
**Texturals features of [32]**		
20	Inverse difference	$\sum_{i=1}^{N_{G}}\sum_{j=1}^{N_{G}}\frac{P\left(i,j\right)\;}{1+\left|i-j\right|}\;$

**Table 3:** Features selection.

Variables	Features Selection				
	Variance Threshold = 0.02	Chi-Squared Test k = 5	SGD Classifier Threshold = ‘Mean’	RFECV	ANOVA *p* Values < 0.05
Extent	X			X	X
Area	X	X	X	X	X
Height	X		X	X	X
Weight	X	X	X	X	X
Compacity	X			X	X
Perimeter	X	X		X	X
Contrast (LBP)	X		X	X	X
Correlation (LBP)	X	X		X	X
Energy (LBP)	X		X	X	X
Homogeneity (LBP)	X		X	X	X
Entropy (LBP)	X		X	X	X
Mean GABOR	X		X	X	X
Standard Deviation GABOR	X			X	X
Dissimilarity (GLCM)	X			X	X
Correlation (GLCM)	X		X	X	X
Contrast (GLCM)	X	X	X	X	X
Homogeneity (GLCM)	X			X	X
ASM (GLCM)	X			X	X

**Table 4:** Model performance.

Models	Accuracy	Precision	Recall	F1-Score
KN	0.80	0.801	0.800	0.799
SGD	0.79	0.794	0.793	0.794
SVM	0.87	0.874	0.873	0.874
RF	0.83	0.838	0.837	0.837

## Data Availability

Data are partly presented in the manuscript and partly available from the corresponding author upon reasonable request.

## Abbreviations

CFA	Color filter array
CCM	Co-occurrence matrices
DT	Decision tree
IQR	Interquartile range
KNN	k-nearest neighbors
LDA	Linear discrimination
LED	Light-emitting diode
MLP	Multilayer perceptron
NB	Naive Bayes
PGM	Portable gray map
RFECV	Recursive feature elimination with cross-validation
RF	Random forest
SVM	Support vector machine
